# Bilateral Buccal Mucosa Graft for Urethroplasty: A Versatile Graft

**DOI:** 10.7759/cureus.54715

**Published:** 2024-02-22

**Authors:** Fattirah Auni Fauzi, Mohd Faizal Abdullah, Shaifulizan Abdul Rahman

**Affiliations:** 1 Oral and Maxillofacial Surgery, Hospital Universiti Sains Malaysia, Kota Bharu, MYS; 2 Oral and Maxillofacial Surgery, School of Dental Sciences, Universiti Sains Malaysia, Health Campus, Kota Bharu, MYS

**Keywords:** reconstruction, urethral stricture, urethroplasty, buccal mucosa graft, bilateral

## Abstract

Male urethral stricture is scarring of the urethral tissue that narrows the urethral lumen causing reduced urinary flow. Urethral reconstruction or substitution urethroplasty using oral mucosa graft, especially from the buccal mucosa, is one of the most widely known techniques to manage urethral stricture. However, studies using bilateral buccal mucosa are still limited. Therefore, this study aims to report our experience and technique of bilateral buccal mucosa grafting for urethroplasty. The authors described a 66-year-old man with long-segment urethral stricture that was successfully treated with urethral reconstruction harvested from bilateral buccal mucosa.

## Introduction

There are numerous past research in the literature that describe surgical alternatives for long-segment urethral stricture reconstruction [1]. The most prevalent method for urethral reconstruction graft is the harvesting of buccal mucosa. However, its application can be restricted when dealing with significant strictures due to the limited available amount of graft tissue. Research indicates that to reduce complications, the maximum graft area from each cheek should not exceed 2.5 cm in width and 4 cm in length [2]. To overcome the limitations associated with graft size, it is necessary to harvest bilateral buccal mucosa grafts. However, it is worth noting that there have been reports of complications, such as perioral numbness, pain, motor deficits, restricted mouth opening, and other issues following the harvesting of oral mucosa graft [3]. An alternative approach involves using a penile fasciocutaneous circular skin flap for stricture lengths up to 24 cm. Nevertheless, it is important to consider that this method has shown a 16% stricture rate at five years. Complications, including skin necrosis, penile scarring, and significant loss of sensation, can occur in patients [4]. Alternative options, such as intestinal grafts in substitution urethroplasties, have also been mentioned in the literature [5]. However, complex procedures involving laparotomy and colon resection make this approach impractical. Hence, the success of the grafts varied depending on the harvesting region, and the patient’s concern should be considered before harvesting the grafts.

## Case presentation

**Figure 1:**
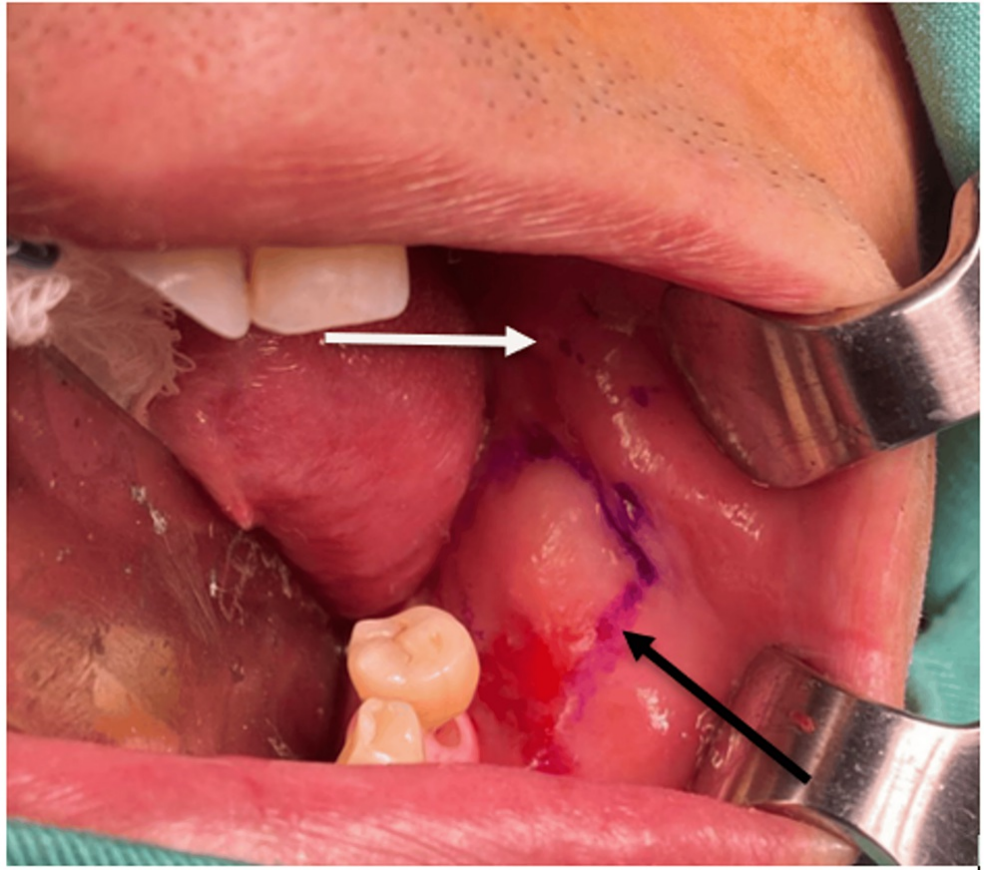
The contralateral oral space was packed with a dry X-ray-detectable gauze sponge. Here, the Stenson’s duct opening was marked. The Army-Navy retractors were only employed in respect of photographing the harvest site. It is commonly not required during harvest. White arrow: Stenson's duct opening marking, black arrow: buccal mucosa graft marking

A 66-year-old man was referred to the Oral and Maxillofacial Unit at Hospital Universiti Sains Malaysia to assess his oral cavity status for bilateral buccal mucosa graft harvest by the Urology Department in August 2020. The patient was finally ready for urethroplasty after he was diagnosed with urethral stricture a few years back. Since then, he has been on a suprapubic catheter. The patient himself could not recall the event that led to the stricture of his urethra, but according to his wife, the history of previous trauma might be the cause of the urethral stricture. He was well aware of the surgical procedures that would be performed as the urologist already informed him regarding possible grafts that could be used for his urethral stricture repair, including a full-thickness skin graft, mucosal colon, and oral mucosa grafts. After explaining the procedures of buccal mucosa graft harvesting, the patient consented to undergo the procedure primarily due to the main advantage of using buccal mucosa grafts, which leaves no visible scar. Upon examination, the patient’s mouth opening was measured at 39 mm with patent bilateral Stenson's duct opening and upper arch and lower arch partial edentulism. Bilateral marking for buccal mucosa graft harvest was performed after cannulation of the bilateral Stenson’s duct using a lacrimal probe. Intraoperative marking for the left donor site is illustrated in Figure 1.

**Figure 2:**
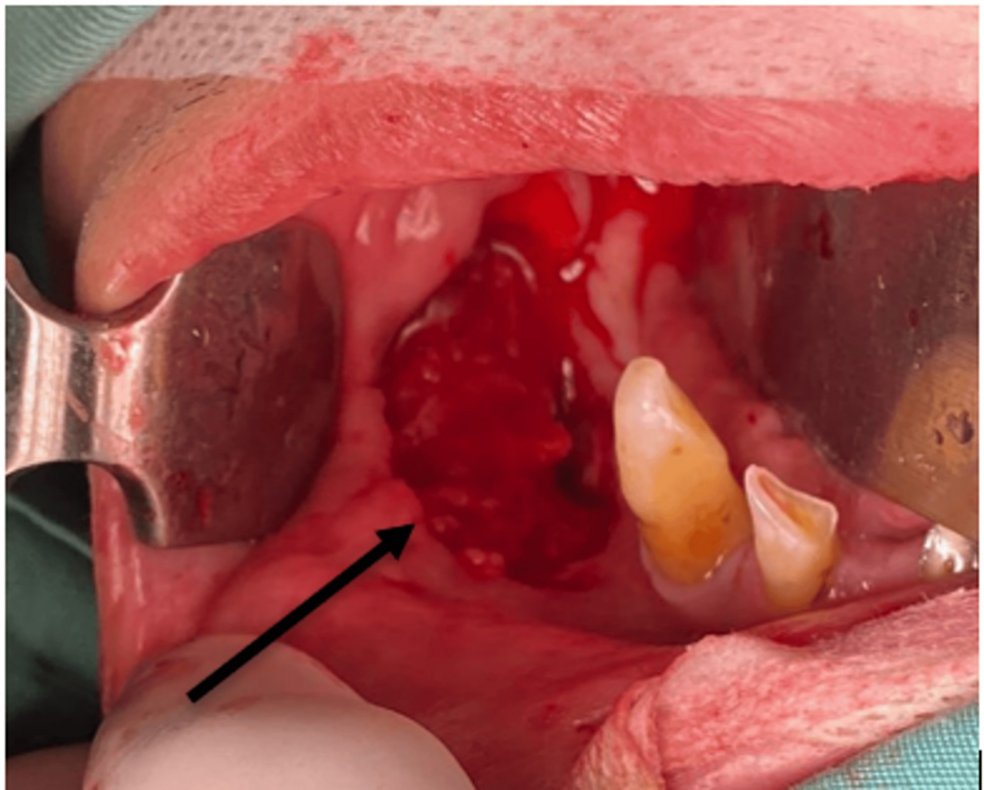
Right donor site defect after the buccal mucosa was harvested. Note that there was a modification for the inferior limb of the buccal mucosa graft, the marking was done close to the lower buccal sulcus to maximize the size of the graft.

In this case, three retraction sutures were employed, with the central suture being integrated into the distal apex of the graft. Subsequently, the right donor site defect after the buccal mucosa graft was harvested is displayed in Figure 2.

**Figure 3:**
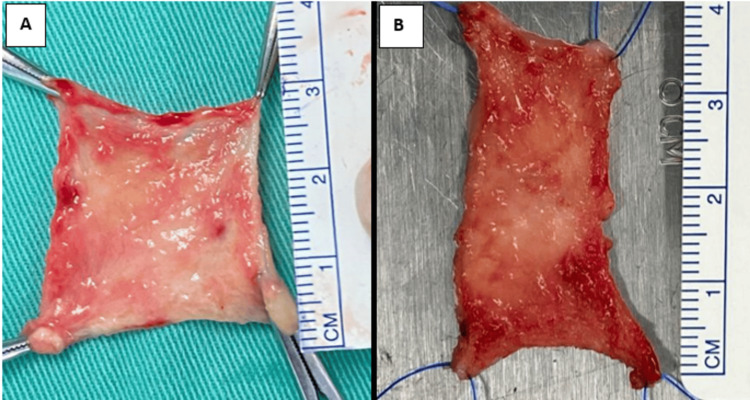
Right (A) and left (B) harvested buccal mucosa graft.

The final harvested buccal mucosa for the right and left side, measuring 3 x 3 cm and 4 x 2 cm, respectively, are presented in Figure 3.

**Figure 4:**
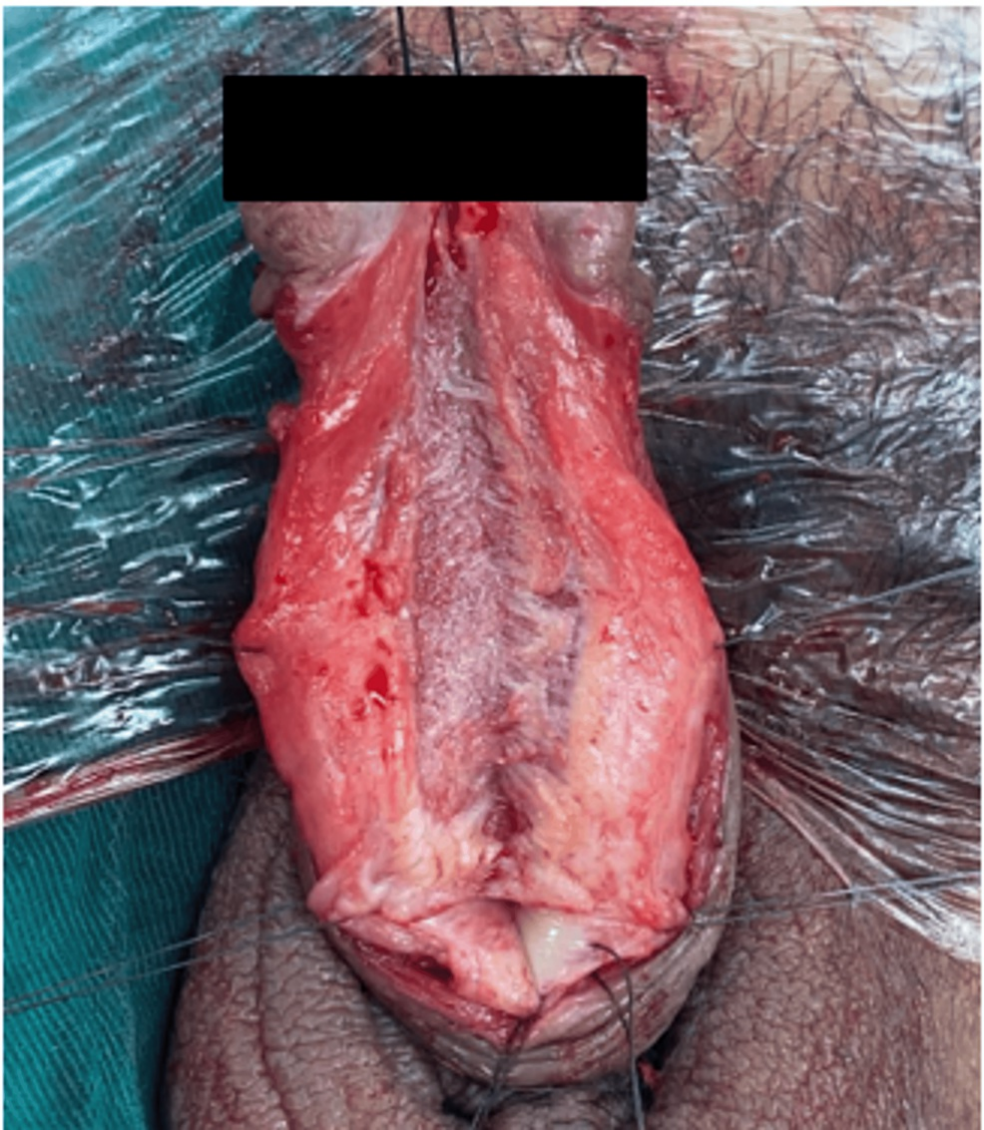
Urethral stricture release and ready for the onlay of the buccal mucosa graft.

The defect was closed primarily without tension with resorbable suture Vicryl size 3/0. Consequently, the grafts were prepared for transfer to the recipient site. The length of the urethra defect was measured and shown in Figure 4, where buccal mucosa graft onlay and suturing were done.

**Figure 5:**
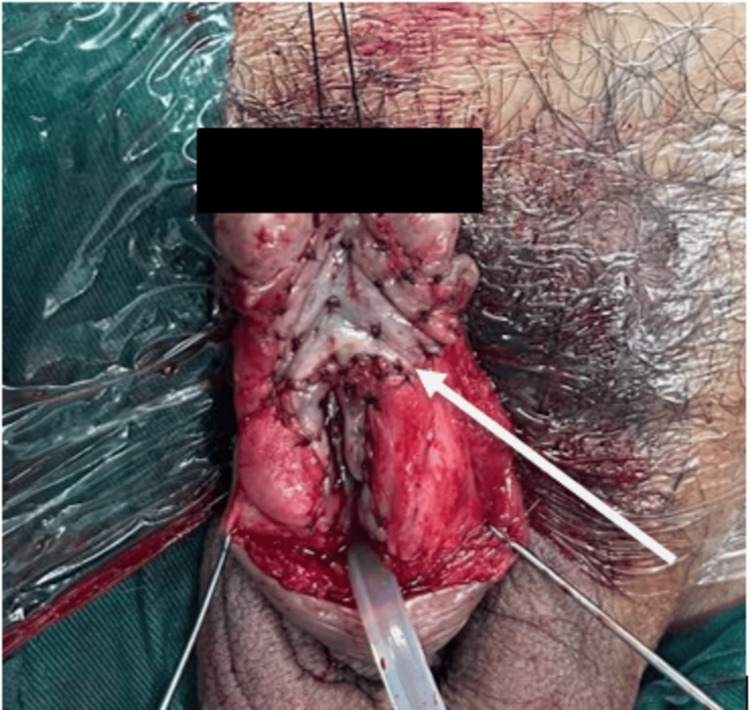
Sutured bilateral buccal mucosa onlay graft.

Complete urethroplasty with bilateral buccal mucosa graft is shown in Figure 5.

**Figure 6:**
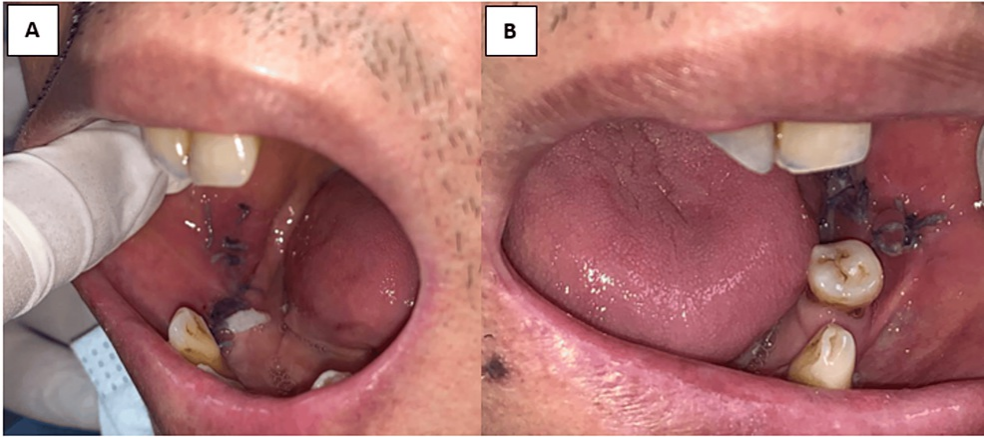
Right (A) and left (B) buccal mucosa appearance on day 5 postoperative with suture in situ.

The patient was reviewed on postoperative day 5, with no paresthesia of the bilateral inner cheek, mouth opening of 39 mm, and both Stenson's ducts patented. The appearance of the donor sites after day 5 postoperative is portrayed in Figure 6.

After the patient was discharged from the ward, he was reviewed periodically up until three years of follow-up. Upon the three-year mark, it was revealed that there were no complications from the donor site. The patient was extremely happy with the outcome of the surgery.

## Discussion

Potential adverse effects of harvesting buccal mucosa include postoperative infection, intraoperative hemorrhage, swelling, pain, restricted oral opening, damage to the parotid duct, and altered sensation over the lower lip or cheek due to nerve damage [6]. Paraesthesia, which involves an abnormal sensation, such as numbness, is the most frequently encountered complication, and it tends to be temporary for most patients. According to Dublin and Stewart’s research [7], 57% of patients experienced numbness after the surgery, and for up to 16% of them, this sensation could persist for as long as a year. Moreover, Caldamone et al. [8] documented two cases of scar contractures resulting from buccal mucosa harvest.

The biological properties with respect to the oral mucosa graft contribute to its success in urethral surgery. When harvesting tissue, it is crucial to take into account anatomic landmarks to ensure optimal patient treatment and reduce donor site morbidity. The versatility of this approach is evident through its extensive application in reconstructing various urethral defects, such as strictures, epispadias, and hypospadias [9-11]. For a graft tissue to be effective, it must fulfill certain criteria: ample tissue availability with minimal morbidity at the donor site, proper adherence of the graft to a vascular bed, and ease of replication and harvesting. The buccal mucosa graft possesses favorable characteristics, including a thick epithelium with a high elastic fiber content, a thin lamina propria, and abundant availability with simple harvest and low morbidity. In addition, the buccal mucosa, being a non-keratinizing stratified squamous epithelium, closely resembles the appearance of the penile and glandular urethra [12].

Once the urologist identifies the necessary graft size, either via preoperative urethrography or intraoperative defect measurement, they utilize a surgical marking pen to delineate the graft’s extent and shape. Consequently, the marking is kept a few millimeters below the papilla of the Stenson’s duct orifice in the buccal harvest site. It is crucial to consider the anticipated graft shrinkage, as research indicates that oral mucosal grafts may reduce in size by up to 20% from their original dimensions during harvest [13]. Oral mucosa serves as a suitable substitute for strictures in the urethra. In addition, it possesses unique immunological properties, and preclinical studies indicate that it displays fibroblast behavior leading to less fibrosis compared to the skin and exhibits a distinct profile from the skin [14]. However, there are specific situations where harvesting buccal mucosa graft is not feasible or recommended, such as in the cases of oral leukoplakia, inadequate oral hygiene combined with heavy tobacco chewing/smoking, prior irradiation, and previous buccal mucosa graft procedures [15].

Even though buccal mucosa graft yields scarless outcomes, the bilateral harvest of the buccal mucosa shows more patient dissatisfaction as compared to a single cheek harvest [16]. Therefore, proper surgical planning and thorough anatomical knowledge will affect the outcome of surgery. As in our patient, even though bilateral buccal mucosa harvest was done, no complications occurred even after three years of follow-up.

## Conclusions

Urethral reconstruction using buccal mucosa graft is widely known in reconstructive world. In order to accommodate significant defects of the urethra, utilization of bilateral buccal mucosa can be implemented. The process of harvesting buccal mucosa graft from both sides of the cheek and subsequently closing the wound is considered a safe procedure and is associated with a high level of patient satisfaction.
